# Influence of antiTNF-alpha antibody treatment on fracture healing under chronic inflammation

**DOI:** 10.1186/1471-2474-15-184

**Published:** 2014-05-29

**Authors:** Melanie Timmen, Heriburg Hidding, Britta Wieskötter, Wolfgang Baum, Thomas Pap, Michael J Raschke, Georg Schett, Jochen Zwerina, Richard Stange

**Affiliations:** 1 Department of Trauma, Hand and Reconstructive Surgery, University Hospital Muenster, Muenster, Germany; 2 Institute for Experimental Muskuloskeletal Medicine IEMM, University Hospital Muenster, Muenster, Germany; 3 Department of Internal Medicine 3, Rheumatology and Clinical Immunology, University of Erlangen-Nuremberg, Erlangen, Germany; 4 Ludwig Boltzmann Institute of Osteology at the Hanusch Hospital of WGKK and AUVA Trauma Centre Meidling, 1st Medical Department, Hanusch Hospital, Vienna, Austria

**Keywords:** Anti-TNFα, Inflammation, Fracture healing, Rheumatoid arthritis, Treatment

## Abstract

**Background:**

The overexpression of tumor necrosis factor (TNF)-α leads to systemic as well as local loss of bone and cartilage and is also an important regulator during fracture healing. In this study, we investigate how TNF-α inhibition using a targeted monoclonal antibody affects fracture healing in a TNF-α driven animal model of human rheumatoid arthritis (RA) and elucidate the question whether enduring the anti TNF-α therapy after trauma is beneficial or not.

**Methods:**

A standardized femur fracture was applied to wild type and human TNF-α transgenic mice (hTNFtg mice), which develop an RA-like chronic polyarthritis. hTNFtg animals were treated with anti-TNF antibody (Infliximab) during the fracture repair. Untreated animals served as controls. Fracture healing was evaluated after 14 and 28 days of treatment by clinical assessment, biomechanical testing and histomorphometry.

**Results:**

High levels of TNF-α influence fracture healing negatively, lead to reduced cartilage and more soft tissue in the callus as well as decreased biomechanical bone stability. Blocking TNF-α in hTNFtg mice lead to similar biomechanical and histomorphometrical properties as in wild type.

**Conclusions:**

High levels of TNF-α during chronic inflammation have a negative impact on fracture healing. Our data suggest that TNF-α inhibition by an anti-TNF antibody does not interfere with fracture healing.

## Background

Inflammatory diseases such as rheumatoid arthritis (RA), do not only increase the risk of fractures [1,2] but may also impair fracture healing by delaying the process and leading to non-unions [3]. Tumor necrosis factor alpha (TNF-α) is one of the main trigger of chronic inflammation in rheumatoid arthritis [4]. TNF-α is also critical for the cause of systemic as well as local loss of bone and cartilage during the course of disease [5]. The use of TNF-α blocking antibodies ameliorates the symptoms of this disease [6]. For instance, treatment with Infliximab, a (chimeric) monoclonal TNF-α antibody, has reduced the symptoms of RA patients [7]. Moreover, TNF-blocking agents combine a strong anti-inflammatory potential leading to direct protection of bone and cartilage [8].

TNF-α is also an important regulator of fracture healing [9]. Aside from Interleukin (IL)-1, -6 and -11, TNF-α is active within the initial inflammatory phase of fracture healing in macrophages and other inflammatory cells, where it leads to neo-angiogenesis and induces osteogenic differentiation of mesenchymal stem cells. In the terminal remodeling phase of fracture healing, high expression of TNF-α and IL-1 activates osteoclasts which degrade the trabecular bone and osteoblasts which regenerate the lamellar bone [10]. Previous studies have demonstrated that lack of TNF-α signaling during fracture healing impairs callus remodeling [11]. Thus, the TNF-α receptor knockout mice show a delay in fracture healing caused by a retarded development of cartilage, followed by chondrocyte apoptosis and remodeling of mineralized cartilage in the late phase of fracture healing [12].

Therefore, TNF-α is an important mediator during different phases of fracture healing. However, the influence of TNF-α blockade, as in treatment of RA patients under chronic inflammatory conditions, is still unknown. A retrospective study of rheumatoid patients treated with TNF- α antagonists showed no decreased risk of fractures [13]. Since TNF-α antibody therapy is widely used for treatment of RA and chronic inflammation, the question remains, whether the therapy should be continued in the case of a fracture or should be suspended. Therefore, we investigated the influence of TNF-α inhibition on fracture healing in an established model of chronic murine rheumatoid/inflammatory arthritis.

## Methods

### Mice and fracture model

**Figure 1:**
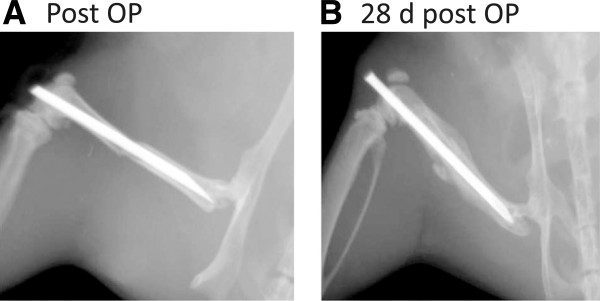
**Radiographs of a fractured femur after surgery OP (A) and after 28 days of healing (B).** Mouse femur fractured and stabilized by an intramedullary nail (Schmidmaier *et al.*, [18], modified).

Generation of heterozygous human tumor necrosis factor transgenic (hTNFtg) mice (strain Tg197) were described previously [14]. Homozygous hTNFtg mice develop a chronic inflammatory arthritis due to the overexpression of human TNF which is acting on the murine TNF receptor I. Disease starts at the age of 6 weeks and is accompanied by local and systemic bone loss reflecting inflammatory bone disease of human rheumatoid arthritis. We used 12 week old, female mice for the fracture experiments. Three groups of 20 mice, including wild type, hTNFtg untreated and hTNFtg treated with a (chimeric) antiTNF-α antibody (Infliximab, 10 mg/kg, 3 times weekly, Centocor, The Netherlands, TNFi) as described [15]. After anaesthesia using a ketamine hydrochloride/xylazine mixture (80 and 12 mg/kg body weight, i.p.) the left leg was fractured with three point bending. It was stabilized with an intramedullary nail (hollow needle 23G) [16] (see also Figure 1). Carprofen (4 mg/kg intra muscular) was given as an analgesic and further on at 24 hour intervals when required. Mice were euthanized by cervical dislocation 14 or 28 days after surgery. All experiments were performed according to the protocol approved by the Animal Care and Use Committee of the University Hospital Erlangen, Germany.

### Biomechanical analyses

For biomechanical analyses, mice (n = 10, each wild type, hTNFtg or hTNFtg treated with Infliximab) were euthanized 28 days after surgery. Both femurs were dissected and prepared for biomechanical testing as described previously [17,18]. Briefly, the proximal and distal ends of each femur were embedded into two molds with bone cement (Palacos R, Heraeus Kulzer GmbH, Germany). Each mold was then connected to a pivoted axis. A linear constant feed rate, generated by a material testing machine (LR5Kplus, Lloyd Instruments, Meerbusch, Germany), was loaded by a lever attached to one of the pivoted axes. The bone was preloaded at an axial force of 5 N and a constant linear propulsion (v = 1 mm/min) was applied by the testing machine. The translation of the material testing machine was transformed to an uniform torsional movement. The free axis was connected to a strain-gauge (*F*_max_ = 250 N, XLC, Lloyd Instruments, Meerbusch, Germany) that detected the torsional force. The data were recorded using Catman32 software (HBM, Darmstadt, Germany) and analyzed by Microsoft Excel (Redmont, USA). The angle of failure and the maximum load were determined as material characteristics and the torsional stiffness (N/°) was calculated as a biomechanical parameter. Each parameter was compared with the values of the non-fractured contralateral femur (ratio of fractured/non-fractured femur (100%)). Data shown are medians with a range of up to 8 measurements for each group.

### Histomorphometry

**Figure 2:**
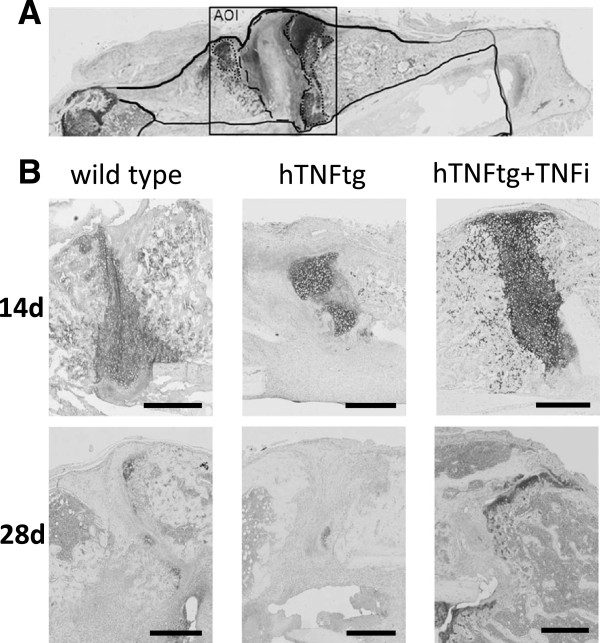
**Histological analysis of the fracture callus composition 14 and 28 days after surgery.** Alcian blue staining was performed using sagittal section of the fractured femur. Using digital image analysis, the area of different tissues was determined within the whole callus as indicated in **A**. Overview of the tissue composition of a sagittal section of a fractured femur: Area of whole callus, black line; cartilage area, dotted line; soft tissue, dashed line. **B**: Comparison of the callus composition after 14 and 28 days with wild type, hTNFtg and hTNFtg + TNFi group within the site of fracture (AOI) after Alcian blue staining (representative pictures, scale bar 500 μm).

For histomorphological analyses fractured femurs (n = 10, each group) were embedded undecalcified in methylmethacrylate (Technovit® 9100, Heraeus, Germany). Sagittal serial sections (5 μm) were stained with alcian blue. Image Pro Plus software (ImagePro®Plus 7.0, Media Cybernetics Inc., Bethesda, USA) was used to determine and calculate the following histological parameters within the area of interest (see also Figure 2A): whole callus area (black line, cartilage area (dotted line)/total callus area and area of soft tissue (dashed line)/total callus area. The histological analysis was performed randomized and blinded with regard to genotype and treatment. Complicated fractures were excluded from the analysis. The data shown are means with SD of up to10 measurements of each group.

### Statistic

Statistical analysis (Mann–Whitney-U-test: conducted between each group, non-parametric, two tailed, Confidence Interval: 95%) was carried out using GraphPad Prism 5.02 (GraphPad Software, San Diego, USA). p-values >0.05 were regarded as significant, p-values between 0.05 and 0.1 indicate tendency.

## Results

### Clinical assessment

As described previously, the hTNFtg mice are smaller in size and weight compared to their wild type littermates [14,19]. A treatment of these mice with a human anti-TNF antibody prevents the development of an inflammatory polyarthritis [14]. In our study, we used hTNFtg mice at the age of 12 weeks for surgery and monitored the follow-up of the healing process for up to 4 weeks. However, the animals were smaller compared to wild type mice. This influenced the outcome of fracturing and therefore the progression of fracture healing. As shown by radiographic and histological pictures, some fractures occurred near the knee joint or were dislocated or fragmentary, and were therefore excluded from further analyses.

### Biomechanical bone stability after fracture

**Figure 3:**
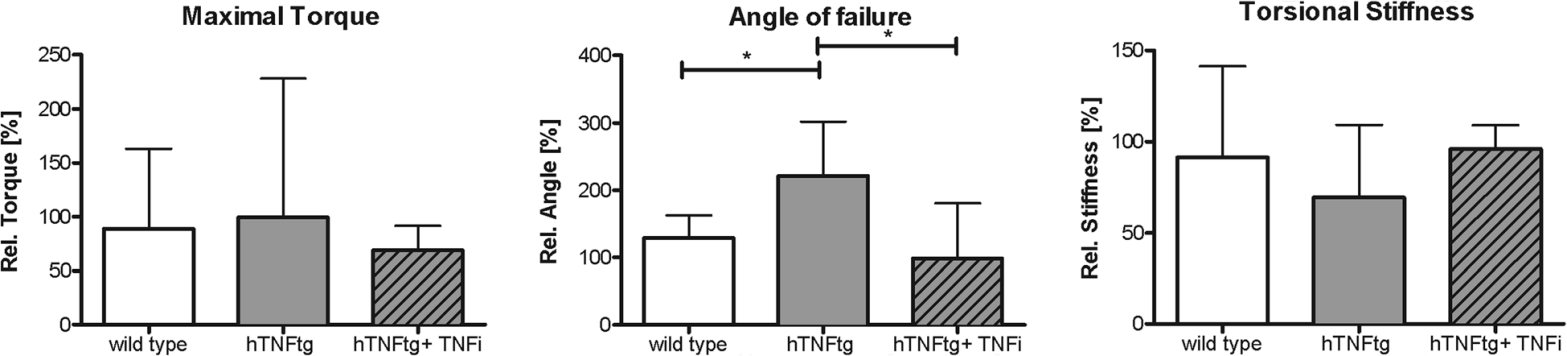
**Biomechanical analysis of the fractured femur.** Torsional testing was applied to the fractured and unfractured femur of each animal. Maximum torque [Nmm] and angle of failure [°] were determined and used for calculation of torsional stiffness [Nmm/°]. The ratio of fractured femur/non-fractured femur [%] was calculated. Wild type: n = 8, hTNFtg: n = 6, hTNFtg + TNFi: n = 7, * p < 0.05; dashed line: p < 0.1, tendency.

Two different material characteristics were measured, that are influenced by the tissue composition during fracture healing. Firstly, the force that is needed to break the bone, characterized by maximum torque, as well the angle of failure that is used as a parameter of the flexibility of the bone, were measured. Secondly, the torsional stiffness was calculated from these values as a measure for the biomechanical stability of the bone. After 28 days of healing there was no significant difference in the maximum torque between the wild type group and both hTNFtg groups, although the standard deviation was high in the hTNFtg group without treatment (Figure 3). The fractured femur yield almost 100% of the maximum torque measured for the unfractured femur in all groups (wild type: 89%, hTNFtg: 99.5%, hTNFtg + TNFi: 69%). Interestingly, the angle of failure in the wild type group (128%) was comparable to the hTNFtg + TNFi group (98%), but significantly increased in the hTNFtg group (221%, wild type vs. hTNFtg: p = 0.0293; hTNFtg vs. hTNFtg + TNFi: p = 0.014). The torsional stiffness, however, was not significantly altered in the hTNFtg group (69.3%) compared to both wild type (91.3%) and the TNFi treated hTNFtg group (96%) (wild type vs. hTNFtg: p = 0.345; hTNFtg vs hTNFtg + TNFi: p = 0.617).

### Histomorphological analysis of fracture callus composition after 14 and 28 days of healing

**Figure 4:**
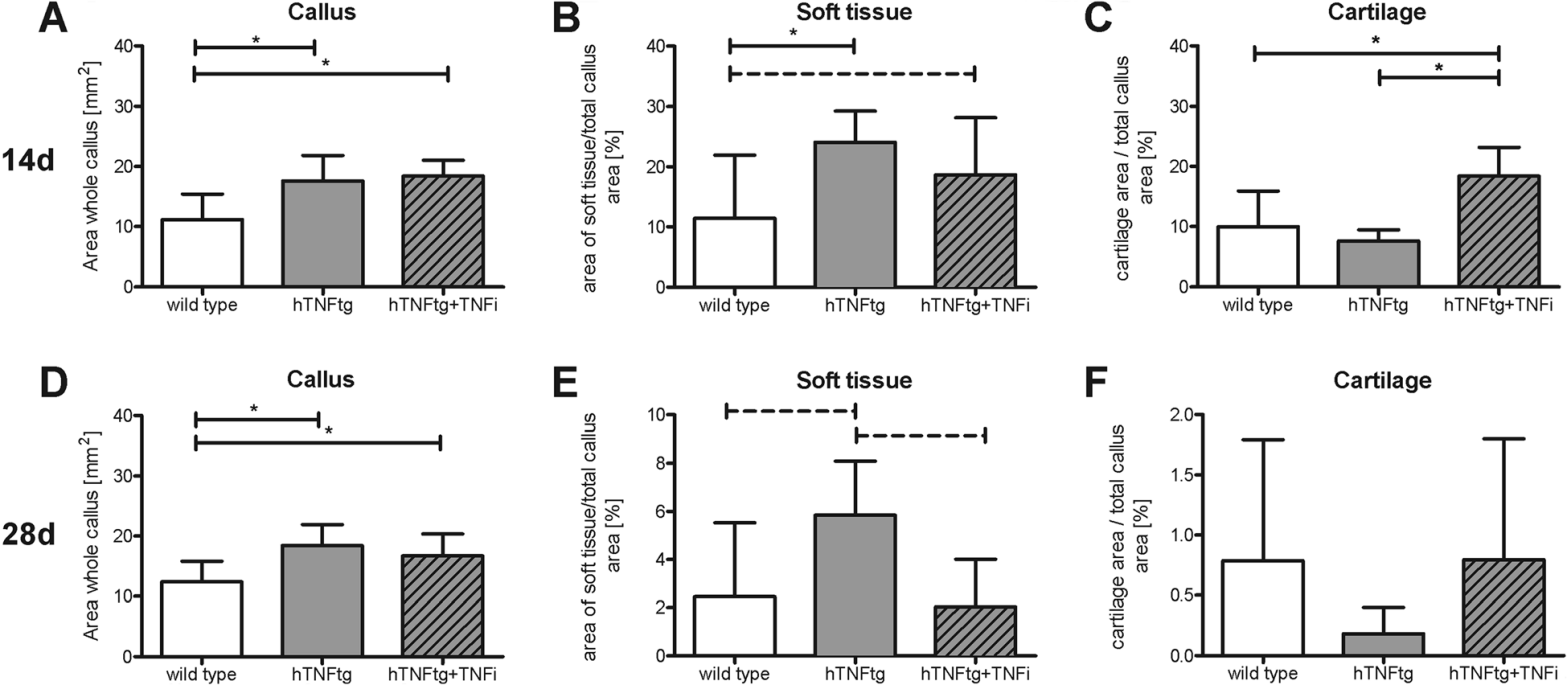
**Quantification of the callus composition after 14 and 28 days of fracture healing using digital image analysis. A/D**: Whole callus area was determined [mm^2^]. Area of soft tissue **(B/E)** as well as Cartilage area **(C/F)** were measured as depicted in Figure 3A and displayed as a ratio to the whole callus area [%]. Wild type: n = 10; hTNFtg: n = 5; hTNFtg + TNFi: n = 7, * p < 0.05, dashed line p < 0.1, tendency.

Further investigation of the callus tissue composition provided an indication for the altered biomechanical properties of the three different groups. Sagittal sections of femurs were stained for cartilage using alcian blue staining and for determination of the whole callus area (Figure 2A). The area of cartilage (stained in blue; dotted line) and the area of soft tissue (dashed line) were compared in the callus area. These parameters change the most within the investigated time points at day 14 and day 28 (Figure 2). The quantitative analysis of all fracture calli showed a significantly bigger callus area in the hTNFtg and hTNFtg + TNFi treated groups compared to the wild type group after 14 days (37%, Figure 4A) and 28 days (30%, Figure 4D). Further investigation of the composition surrounding the fracture site revealed an increased area of soft tissue in the hTNFtg group (24.1% vs. 11.4% (wild type, p = 0.0307) and 18.7% (hTNFtg + TNFi) compared to the wild type at day 14 (Figure 4B). Additionally, the hTNFtg + TNFi group displayed twice as much cartilage than both other groups on day 14 (18.4% vs 9.9% in wild type (p = 0.0234) or 7.6% in hTNFtg (p = 0.0159) (Figure 4C). After 28 days the content of soft tissue was higher within the wild type group (5.8% vs. 2.5% in wild type and 2% in hTNFtg + TNFi; p < 0.1). The amount of cartilage in wild type and hTNFtg + TNFi group was lower (0.8%) compared to 14 days, but much less in hTNFtg without treatment (0.18%) (Figure 4B). Furthermore, the area of mineralized bone (trabecular bone within the callus area) was determined, but there were no differences within the three groups (Data not shown).

## Discussion

The hTNFtg mouse is considered to be a valuable model of human RA [19,20]. In these animals, overexpression of human TNF-α induces severe inflammatory polyarthritis. This arthritis shares many hallmarks with the human counterpart, such as polyarticular involvement of the small joints, chronic and progressive course of disease, and the destruction of cartilage and subchondral bone. The disease is driven by the overexpression of proinflammatory cytokines. The overexpression of TNF-α was described as being responsible for a dramatic loss of trabecular bone and, to a lesser extent, cortical bone [20]. The influence of a chronic inflammation on fracture healing, however, has not been studied. Therefore, we used hTNFtg animals, with high systemic levels of TNF-α to investigate its influence on the composition of the fracture callus and the progression of the healing process.

The importance of TNF-α signaling during fracture healing is commonly accepted today. On the one hand, it is known that TNF-α expression increases soon after injury [11,21]. During this inflammatory phase different cell types, including mesenchymal stem cells, located in the surrounding tissue, are recruited to the fracture site and initiate the formation of the primary callus. Different studies were able to demonstrate that deficiency of TNF-α signaling causes a delay in callus remodeling with regard to chondrocyte maturation and apoptosis in the later phases of fracture healing [11,12,22]. On the other hand, high levels of TNF-α contribute to accelerated loss of cartilage during fracture healing as seen in diabetic mice [23]. In our study we have demonstrated that high systemic levels of TNF-α influence the biomechanical stability and composition of the bone in the course of fracture healing, lead to increased callus formation with less cartilage and more soft tissue within the fracture gap. These alterations of the callus composition resulted in lowered biomechanical stability with a higher flexibility of bone within the callus after 28 days of healing. Histologically, we found that the callus of hTNFtg animals was built of less cartilage and more soft tissue compared to the wild type which leads to an altered stability of the callus. Therefore, our results indicate a negative influence of chronically elevated TNF-α level during the fracture healing process.

TNF-α-inhibitors like Infliximab are used in treatment of rheumatic patients to bind TNF-α with high affinity to reduce inflammatory symptoms [6,24]. The high efficiency of the blocking hTNF antibody was proven within the hTNFtg animal model [14]. Treated animals do not show any signs of rheumatoid like disease. The influence of a TNF-α-inhibitor on fracture healing, however, has been investigated in a wild type rat model under non-inflammatory conditions. In this case the authors did not find any impairment of metaphysal bone healing [25]. Under chronic inflammatory conditions, as it was shown in this study, the blockade of TNF-α during fracture healing (by Infliximab treatment) led to an increased callus size, which was accompanied by an increase in cartilage tissue compared to the not treated group. After 28 days of fracture healing, the fracture callus composition resembled the wild type situation. Biomechanical testing revealed no differences compared to wild type fracture healing. Treatment with Infliximab even seemed to compensate the derogating effects of elevated systemic TNF-α levels on fracture healing which can be concluded from the similarity of the biomechanical and histological results of the TNFi treated group compared to wild type in our model. It might be interesting to further distinguish between the negative effects of pathologically elevated levels of TNF-α and the positive effects of endogenous TNF signaling during fracture healing. This could be done by performing studies using the fracture model and dose dependent treatment with hTNF blocking antibody and to look for the onset of callus development and callus composition in the course of healing. Our results provide indication that rheumatoid arthritis patients, who sustained a fracture during the treatment with TNF-α antagonist, should continue this therapy.

## Conclusion

Elevated TNF-α level have a negative impact on the physiological development of the callus and thereby impair fracture healing in this murine model of chronic inflammation. We have found that the treatment of this chronic inflammation during fracture healing is beneficial and restores the biomechanical and morphological condition as found in the wild type. Further studies on fracture healing under chronic inflammatory conditions in animal models and rheumatoid arthritis patients will be necessary to further elucidate the mechanisms of this positive effect of anti-TNFα-treatment.

## Abbreviations

TNF-α: Tumor necrosis factor alpha; RA: Rheumatoid arthritis; hTNFtg: Mice with a human TNF alpha transgenic background; IL: Interleukin; TNFi: AntiTNFalpha antibody treatment; MSC: Mesenchymal stem cell; AOI: Area of interest.

## Competing interests

The authors declare that they have no competing interests.

## Authors’ contributions

MT, WB, HH, BW and RS collected the data. RS, TP and JZ designed the study. TP, MR and GS contributed to the data analysis and interpretation. MT, RS and JZ wrote the manuscript. All authors read and approved the final manuscript.

## Pre-publication history

The pre-publication history for this paper can be accessed here:

http://www.biomedcentral.com/1471-2474/15/184/prepub
